# Biological and immunotoxicity evaluation of antimicrobial peptide-loaded coatings using a layer-by-layer process on titanium

**DOI:** 10.1038/srep16336

**Published:** 2015-11-09

**Authors:** Jue Shi, Yu Liu, Ying Wang, Jing Zhang, Shifang Zhao, Guoli Yang

**Affiliations:** 1 Department of Oral and Maxillofacial Surgery, Stomatology Hospital, School of Medical, Zhejiang university, Yan’an Road, Hangzhou 310006, China; 2 Department of Oral Medicine, Stomatology Hospital, School of Medical, Zhejiang university, Yan’an Road, Hangzhou 310006, China; 3 Department of Implantology, Stomatology Hospital, School of Medical, Zhejiang university, Yan’an Road, Hangzhou 310006, China

## Abstract

The prevention and control of peri-implantitis is a challenge in dental implant surgery. Dental implants with sustained antimicrobial coating are an ideal way of preventing peri-implantitis. This study reports development of a non- immunotoxicity multilayered coating on a titanium surface that had sustained antimicrobial activity and limited early biofilm formation. In this study, the broad spectrum AMP, Tet213, was linked to collagen IV through sulfo-SMPB and has been renamed as AMPCol. The multilayer AMPCol coatings were assembled on smooth titanium surfaces using a LBL technique. Using XPS, AFM, contact angle analysis, and QCM, layer-by-layer accumulation of coating thickness was measured and increased surface wetting compared to controls was confirmed. Non-cytotoxicity to HaCaT and low erythrocyte hemolysis by the AMPCol coatings was observed. *In vivo* immunotoxicity assays showed IP administration of AMPCol did not effect serum immunoglobulin levels. This coating with controlled release of AMP decreased the growth of both a Gram-positive aerobe (*Staphylococcus aureus*) and a Gram-negative anaerobe (*Porphyromonas gingivalis*) up to one month. Early *S. aureus* biofilm formation was inhibited by the coating. The excellent long-term sustained antimicrobial activity of this multilayer coating is a potential method for preventing peri-implantitis through coated on the neck of implants before surgery.

Due to their high success rate and predictability, oral implants are now the preferred method for replacing missing teeth. Fixed and removable dental prostheses can be affixed with the support of implants^1^,^2^,^3^. Although the implant success rate is high, failures due to implant-associated infections do occur, which results in bone loss around the implant and subsequent loss of osseointegration^4^,^5^. Depending on the characteristics and location, this disease occurs in two common forms; peri-implantitis and peri-implant mucositis^6^.

Peri-implantitis was first described as an infectious disease similar to periodontitis by Mombelli *et al.* in 1987^7^. Peri-implantitis occurs in 28% to 56% of affected patients and in 12% to 43% of individual implants^8^. Evolution of systematic reviews and meta-analyses have allowed precise measurements of peri-implantitis frequencies for different prostheses. Peri-implantitis occurred in 18.8% of patients and in 9.6% of implants in normal and high-risk patients followed over five years^9^. Medium and long-term follow-up studies showed that the peri-implantitis occurred in 10.4% of patients requiring full-arch restorations. Additionally, in 9.1% of patients requiring implant-supported over-dentures, 8% of implants developed peri-implantitis. Overall, 5.4% of implants and 9.4% of prostheses developed peri-implantitis in patients requiring implant-supported cantilever fixed dental prostheses^9^,^10^,^11^,^12^,^13^.

Few treatments exist for peri-implantitis. Therapy for peri-implantitis includes nonsurgical and surgical phases. Mechanical, ultrasonic, or laser-mediated debridement, either alone or in combination with antiseptic and/or antibiotic agents, occurs in the nonsurgical phase, while the surgical phase includes resective or regenerative techniques^14^.

Excessive mechanical stress^15^, poor implant design^16^, corrosion, and some systemic diseases including diabetes mellitus^17^, osteoporosis^18^, and long-term treatment with corticoids, radiation, or chemotherapy^19^ are important risk factors associated with the onset and development of peri-implantitis. Additionally, bacterial infections play an important role in the failure of dental implants and development of peri-implantitis. The microbiota present in peri-implantitis patients is similar to patients affected by advanced periodontitis. High levels of non-motile anaerobic Gram-negative bacteria such as *Porphyromonas gingivalis* and *Aggregatibacter actinomycetemcomitans*^20^ are present in both disease states. However, the peri-implant microflora differs from species found on the surface of teeth. For example, *Staphylococcus aureus*^21^ has been associated with bleeding upon probing and suppuration, likely due to its relatively high level of adhesiveness to titanium surfaces^7^,^22^,^23^.

Bacterial infections on inorganic surfaces in the mouth are difficult to treat because the local drug dose is low following systemic antibiotic administration due to biofilm formation. Local drug-delivery devices may improve antibiotic delivery to the dental implant surface. Lang *et al.*^5^ inserted tetracycline fibers for 10 days in the gingival sulcus surrounding the dental implant, providing a sustained delivery of high dose antimicrobial directly to the affected site. Drug-resistant bacteria pose another challenge in the use of conventional antibiotics, especially multi-drug resistant bacteria such as methicillin-resistant *S. aureus* (MRSA)^24^,^25^. Hence, the ideal delivery mechanism must provide specifically localized antimicrobial activity without increasing antibiotic resistance.

Cationic antimicrobial peptides (AMP) are non-classical drugs which form a central component in the defense mechanisms of all species in nature^26^,^27^. AMPs are short (12–50 amino acids) cationids (due to lysine and arginine residues) that have broad-spectrum bactericidal activity to Gram-positive (G+) and Gram-negative (G−) bacteria^28^,^29^,^30^. Recently, new synthetic AMPs with improved antimicrobial activity have been developed using native AMPs as templates^31^,^32^,^33^,^34^.

The exact mechanism of AMP antimicrobial activity is not clear. Hilpert *et al.*^35^ demonstrated that AMPs have an increased affinity for the negatively charged membranes of bacteria and act either by bacterial membrane permeabilization or by effecting cytoplasmic targets following translocation across the bacterial membrane. A high local concentration of the AMP results in displacement of positively charged counterions attached to the outer surface layers, inducing a change in bacterial surface electrostatics causing lysis. Hancock *et al.*^36^,^37^ suggested that AMPs possess immunomodulatory activity, including the ability to reduce LPS-mediated pro-inflammatory responses.

Calcium phosphate coatings, vertically aligned titanium nanotubes, collagen gels, chitosan coatings, fibrin scaffolds and POPC (palmitoyloleoyl phosphatidyl-choline)^38^,^39^,^40^,^41^ coatings have all been proposed as AMP delivery systems. Each approach has advantages, but all lacked a prolonged exposure rate. Across all methods, the longest exposure was approximately 3 hours, not enough to influence the soft tissue healing and osseointegration that proceed over a much longer time span after implant placement. It needs about one month for soft tissue healing and three months for osseointegration. However, the layer-by-layer assembly (LBL) technique first introduced by Gero Decher in 1977^42^ has emerged as a simple and versatile method for coating biological templates for biomedical applications^43^. Films are formed through attractive electrostatic forces between charged polyanions and oppositely charged polycations. Changing the number of layers can control the desired film thickness. Following degradation of each layer, the antimicrobial agent can be released continuously at a sustained level over time.

We used to layer-by-layer technique on a pure titanium surface as a delivery system for AMPs. Here we present the biocompatibility, erythrocyte cytotoxicity, immunotoxicity (unintended immunosuppression or enhancement), degradation behavior and sustained antimicrobial activity of the delivery system.

## Materials and Methods

### Preparation of AMPCol

**Figure 1:**
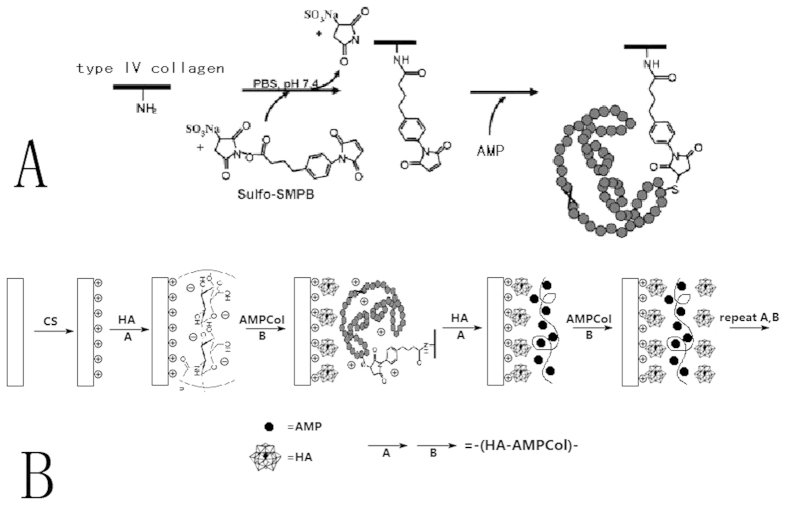
The images showed the establishment of CS-(HA-AMPCol) n coating. (**A**) Synthesis of AMPCol. Sulfo-SMPB was first linked to collagen IV in PBS_7.4_ buffer, then AMP was conjugated with this composite product to obtain AMPCol. (**B**) The CS-(HA-AMPCol) n coating was constructed using the LBL technique.

The antimicrobial peptide, Tet213 (KRWWKWWRRC), was used in this study, and its effective antimicrobial activity has been shown by Wang *et al.*^39^. According to the reaction equation in Fig. 1A, AMP was covalently linked to free amines of collagen IV (Sigma-Aldrich, St. Louis, MO, USA) via sulfosuccinimidyl-4-(p-maleimidophenyl)butyrate (sulfo-SMPB) (Pierce, Rockford, IL, USA). The product was renamed AMPCol, which was provided by the Apeptide Company (Shanghai, PR China).

### Surface Treatment of Titanium Plates

Titanium plates (Guangci Medical Appliance Company, Zhejiang, PR China) 10 × 10 × 1 mm in size were polished to achieve a similar surface texture to transgingival implants. Briefly, samples were polished with silicon carbide (SiC) abrasive paper of various grit sizes (from #320 to #2400), then rinsed with acetone, 75% alcohol, and distilled water in an ultrasonic cleaner. Samples were dried under nitrogen.

### Fabrication of Antimicrobial Peptide Coatings

Solutions of chitosan (CS) (5 mg/ml) (Yunzhou Biochemistry, Qingdao, PR China) in 0.2% acetic acid, hyaluronic acid (HA) (0.5 mg/ml) (Sigma Chemical Co., MO, USA) in distilled water, and AMPCol solution (1 mg/ml) in distilled water were used sequentially for our LBL technique. Polished titanium plates were immersed in the CS solution for 30 min, forming a precursor layer with a stable positive charge, which initiated the layer-by-layer assembly process. Plates were dipped into the HA solution at room temperature for 5 minutes, so HA was adhered electrostatically onto the surface. Plates were then dipped into the AMPCol solution for 5 minutes at room temperature resulting in a positively charged surface. Layering of HA and AMPCol was repeated as desired without drying. We define number of layers as the number of peptide and HA adsorption steps. For example, five layers correspond to the adsorption of CS-(HA-AMPCol)5.

### Degradation Behavior of Multilayer Coatings

To investigate degradation of the coatings, ultraviolet-visible spectroscopy (UV/Vis) was used to measure the absorption peak of AMP at 280 nm^41^,^44^. The titanium plates with CS-(HA-AMPCol)5, CS-(HA-AMPCol)7, or CS-(HA-AMPCol)10 were immersed in 1 ml phosphate buffered saline (PBS, pH 7.4) at 37 °C. At various time intervals, 0.5 ml of solution was sampled and the volume replenished with fresh PBS. Samples were stored at −20 °C. A standard curve was obtained using a series of AMPCol solutions in the concentration range of 0.03125–1.00 mg/ml. The amount of cumulative AMPCol released was measured using a luminometer and calculated based on the standard curve. Each group contained three samples.

### Analysis of Surface Characteristics

X-ray photoelectron spectroscopy (XPS) measurements were performed using an AXIS ULTRADLD spectrometer (VG ESCALAB MARK II) with a monochromatized Al K-alpha X-ray source operating at 15 kV and 8 mA. Contact angle measurements were obtained using a dynamic contact angle system (SL200B, Solon Tech. Inc. Ltd. Shanghai, China). Ultra pure water was used as the wetting agent at room temperature. The mean of at least three independent measurements was reported for each contact angle. Atomic force microscopy (AFM) images were recorded in the tapping mode at 20–25 °C in air with a Seiko SPI3800N station (Seiko Instrument Inc.), using silicon tips (NSG10, NT-MDT) with a resonance frequency of approximately 300 kHz. A quartz crystal microbalance (QCM, Resonance Probe GmbH, Goslar, Germany) was used to measure the thickness of each HA-AMPCol layer. Gold-polished AT-cut quartz crystals (Maxtek) with a fundamental frequency (***ƒ***_***0***_) of 5 MHz and gold electrodes were used. Multilayer coatings were prepared using a similar procedure on the front side of the quartz crystals. The frequency shifts of the quartz crystal, 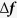, were acquired at third (15 MHz) overtones. Using Sauerbrey’s equation^45^, the areal mass of each layer, 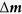, was calculated using the following equation:


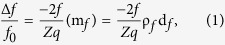


Sauerbrey’s equation. Where **ƒ** = ***nƒ***_***0***_ is the resonance frequency, ***m***_***ƒ***_ is the mass density of the film, 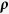
_***ƒ***_is the density of the film and ***Zq*** is the acoustic impedance of crystalline quartz. After applying this calculation, the thickness of each layer, ***d***_***ƒ***_(nm), can be estimated as ***d***_***ƒ***_ = 0.176***n·***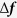, where n is the number of overtones.

### Tensile Bond Strength Test

As the intended location of use is the neck of implant, and this coating is expected to have a strong bond strength with polished titanium, especially in terms of tensile strength (rather than shear strength). Titanium plates with CS-(HA-AMPCol)5, CS-(HA-AMPCol)7 and CS-(HA-AMPCol)10 were tested individually for tensile bond strength^46^ with the help of Zwick Universal testing machine (Z2.5, German) which is digitally controlled and has a software for test control and data acquisition. Two cylinders were used in the test. The samples were bonded to both cylinders. The vertical uniaxial tensile load (20 kN) was applied to the ends of the cylinder with a cross speed of 5 mm/min until debonding of the test material. This force in Newton was recorded by the system. Typical bond strength was calculated in Megapascals, taking into account the surface area of the adhesive interface. Since the test area was a square (10 mm × 10 mm) in shape, the area of the cross section was calculated to be 100 mm^2^. The tensile bond strength was calculated using the following equation:


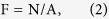


Tensile bond strength calculation equation. Where F is the tensile bond strength (MPa), N is the vertical uniaxial load exerted on the sample (Newton) and A is the size of bonding area, which is 100 mm^2^. All measurements were repeated three times.

### Bio-toxicity Assay

#### Cytotoxicity Assay

HaCaT cells (Cellbank of the Chinese Science Academy, China) were cultured in Roswell Park Memorial Institute-1640 medium (RPMI-1640; Gibco BRL, Grand Island, NY, USA) supplemented with 10% fetal bovine serum (FBS; Gibco BRL, USA) at 37 °C with 5% CO_2_. Media was replaced every 3 days. Cell viability was determined using AlamarBlue cell viability reagent (Invitrogen, Grand Island, NY, USA). Briefly, cells were cultured on the surfaces of titanium plates with different multilayer coatings or control surfaces at a density of 2 × 10^4^/well. Each group contained 6 samples. Culture medium was replaced with fresh medium containing 10% AlamarBlue 4 hours after seeding and incubated at 37 °C for 4 hours. Samples were exposed to an excitation wavelength of 540 nm and emissions at 590 nm were recorded. Using these results, cytotoxic effects of titanium plates with different coatings were measured. Measurements were taken at 24 hours and 3 days and results are expressed as mean values representative of two independent experiments.

#### Erythrocyte Hemolysis Assay

Human erythrocytes were isolated from whole blood taken from healthy donors in the Stomatology Hospital, Zhejiang University School of Medicine, China. Erythrocytes were isolated by centrifugation (1500 × *g* for 10 minutes at 4 °C), washed three times with 0.9% NaCl and finally resuspended in 0.9% NaCl solution to a hematocrit level of 5%. Titanium plates with CS-HA, CS-(HA-AMPCol)5, CS-(HA-AMPCol)7, CS-(HA-AMPCol)10, and pure titanium (negative control) were immersed in 2 ml erythrocyte suspension for 3 hr and 24 hr at 37 °C, respectively. Samples were used for both the hemolysis assay and malondialdehyde (MDA) evaluation. The content of hemoglobin in supernatants was determined spectrophotometrically at 540 nm using the Drabkin’s method^47^. The percentage of hemolysis was calculated using the following formula:


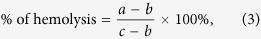


Hemolysis calculation equation. Where ***a*** is the optical density (OD) of the sample at 540 nm, ***b*** is the OD of the control (incubated with PBS), and ***c*** is the OD of 100% hemolysis (incubated with distilled H_2_O).

MDA, the end product of lipid peroxidation (LPO) of the erythrocyte plasma membrane, indirectly reflects the degree of erythrocyte damage. MDA was measured by the thiobarbituric acid (TBA) method using a trace assay kit (Nanjing Jiancheng Bioengineering Institute, Nanjing, China). Briefly, the samples and the appropriate reagents were mixed using a vortex and placed in a 95 °C water bath for 40 minutes. The absorbance was then measured at 532 nm using the spectrophotometer. All measurements were repeated three times.

#### *In vivo* immunotoxicity assay

Animal experiments were approved by the Institutional Animal Care and Use Committee of Zhejiang University, Hangzhou, China. Inbred male BALB/c mice, 6 weeks of age with an average body weight of 18–22 g, were procured from Zhejiang University Experimental Animal Center (Hangzhou, China). Mice were randomly assigned to four groups (five per cage) and acclimatized for 1 week in shoe box cages containing heat-treated pine shavings in rooms maintained at an ambient temperature of 22 °C with a 12 hour/12 hour light/dark cycle. Mice were intraperitoneally injected with AMPCol (2.5 mg/kg/day) or AMP (2.5 mg/kg/day) for 4 weeks (7 days/week). Negative control mice were injected with an equal amount of PBS. Positive control mice were injected with cyclophosphamide (CTX) (250 mg/kg) at 24 hours before the end of the treatment period. Body weight gain was recorded daily. Mice were euthanized by carbon dioxide gas and trunk blood was collected. Spleens were aseptically excised and weighed. The study protocol was reviewed and approved by the Ethics Committee for experimental animals, Zhejiang University (no. 866, Yuhangtang Road, Hangzhou, P.R. China). The methods were carried out in accordance with the ARRIVE guidelines^48^.

Immunoglobulin levels^49^,^50^ were analyzed using a mouse IgM, IgA and IgG enzyme-linked immunsorbent assay (ELISA) Quantitation Kit (IgM and IgG from Abcam, Cambridge, UK; IgA from Sigma, USA) following the manufacturer’s instructions. Briefly, a 96-well plate was coated with affinity purified HRP-tagged mouse Ig (IgM or IgA or IgG) coating antibody. Testing sample was incubated with working assay solution at 37 °C for 15 min on a 96-well plate. The reaction was terminated by the addition of stop solution to each well. The absorbance values of the wells were measured at 450 nm using a spectrophotometer.

Organ relative weight was calculated was calculated using the following formula:


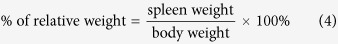


Organ relative weight calculation equation.

#### Culture of Bacteria

Two different types of bacteria were cultured to determine antimicrobial activity. *S. aureus* (ATCC 25923), a Gram-positive aerobe, was grown at 37 °C overnight in Columbia Blood agar (BioMérieux, Marcy l’Etoile, France), while *P. gingivalis* (ATCC 33277), a Gram-negative anaerobe, was grown in an anaerobic system at 37 °C for 7 days in Brain Heart Infusion (BHI) agar (Oxoid, Hampshire, UK). After incubation, a single colony of both bacteria were transferred intoto BHI broth (Oxoid, UK), and incubated either at 37 °C for 1 hour (*S. aureus*) or in an anaerobic at 37 °C for 4 days (*P. gingivalis*), to obtain bacteria in the mid-logarithmic phase of growth. The suspensions were centrifuged at 2,100 rpm for 10 min, and microbial concentration adjusted to a McFarland standard of 0.5 (1.5 × 10^8^ CFU/ml) using a Densicheck (BioMérieux, Marcy l′Etoile, France). Suspensions were used for antimicrobial activity testing.

#### Sustained Antimicrobial Activity Testing

To investigate length of sustained antimicrobial activity, titanium plates with CS-(HA-AMPCol)10 or plates without coating were immersed in BHI broth at 37 °C. Every 4 days, broth with AMPCol was collected by two methods: (1) collecting part of the broth without replenishing, and (2) collecting all broth for analysis then adding an equal amount of fresh broth. Subsequently, 0.25 ml *S. aureus* or *P. gingivalis* suspensions were added to 2.5 ml of testing broth and incubated at 37 °C for 4 hours or in an anaerobic environment at 37 °C for 4 days, respectively. After incubation, the residual bacteria were plated on Columbia Blood Agar and BHI Agar, respectively, and incubated as described above. The survival rate of the bacteria was assessed by counting the number of colony-forming units (CFU). Six samples were analyzed for each time interval, and results are representative of two independent experiments.

#### Early Biofilm Formation and Fluorescence Microscopy

Early biofilm formation of *S. aureus* was assessed using the method from Palestrant *et al.*^51^. Titanium plates with CS-(HA-AMPCol)n or control plates were immersed in 1 ml BHI broth separately, and 0.1 ml *S. aureus* suspension was added. After 90 minutes of biofilm adhesion, each plate was washed with 10 ml PBS to remove unattached bacteria, and then fixed in 2.5% glutaraldehyde at 4 °C for 1 hour. After fixation, the biofilms were stained with a 0.01% acridine orange (Sigma, USA) solution, and washed 3 times with PBS. Biofilms were observed using a Nikon Eclipse 80i fluorescence microscope with an argon laser at an excitation wavelength of 488 nm (green fluorescence). Images were captured using Nis-Elements AR software (Nikon, Tokyo, Japan). Fluorescence intensities were quantitatively analyzed using Image-Pro Plus 6.0 (Media Cybernetics, USA) and expressed by mean optical density (MOD), which was calculated through integrated optical density (IOD)^52^. For each group, the average values MOD were calculated from the images taken by the CCD camera. Results are representative of two independent experiments of six samples per condition.

#### Statistical Analysis

Group means and standard deviations were calculated for each parameter. Data were tested for normal distribution and statistically analyzed using one-way analysis of variance (one-way ANOVA). IBM SPSS Statistics software 19.0 (Statistical Package for the Social Sciences, SPSS) was used for all statistical analyses. P values of <0.05 were considered significant.

## Results

### Development of an AMPCol surface through the layer-by-layer technique

Multilayered AMPCol coatings were fabricated on the polished titanium plates using the LBL technique as described by Liu *et al.*^39^ and Jiang *et al.*^40^ with some modifications as shown in Fig. 1B. The isoelectric points of collagen IV and HA are 7.5–8.0^41^ and 2.9^42^, respectively. When dissolved in a solution at pH 5.0, collagen IV can carry a positive charge, while HA carries a negative charge.

**Figure 2:**
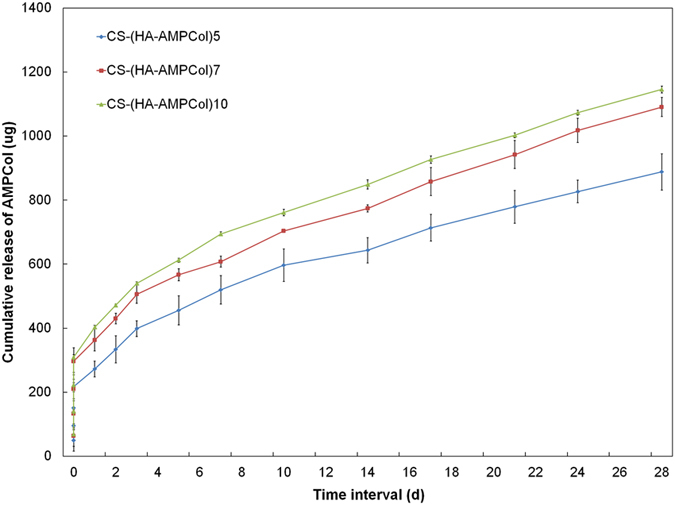
The kinetics of AMPCol release into PBS from CS-(HA-AMPCol)n titanium surface at different degradation times. Data shown are mean ± SD of a single experiment (n = 3 for each group), which was repeated three times.

As shown in Fig. 2, AMPCol release increased as assembly layers increased and was released at a steady rate over time, through 28 days of measurement.

**Figure 3:**
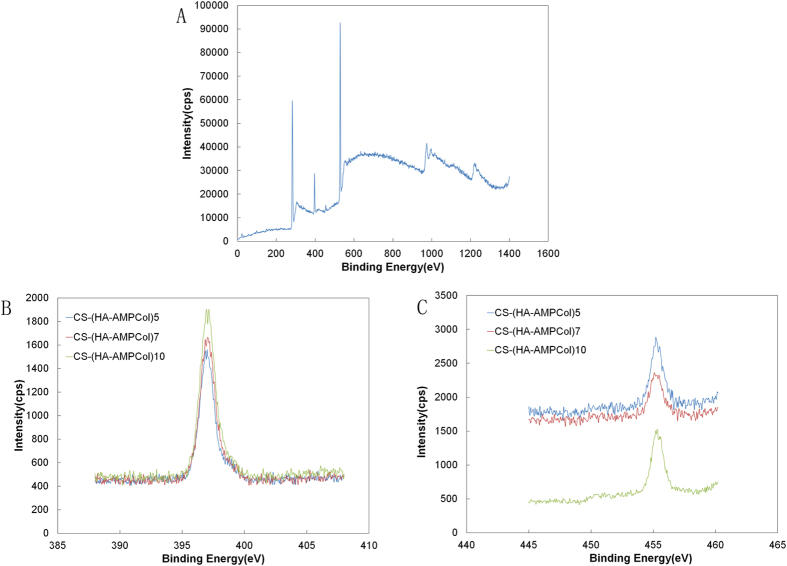
XPS spectra of titanium surface coated with CS-(HA-AMPCol)10 coating showed the elements in this coating: (**A**) XPS spectra; (**B**) N 1s spectra; (**C**) Ti 2p spectra.

We used XPS analysis to determine the adsorption of polyelectrolytes on the coated titanium surface. Signals corresponding to Ti, O, C, N and S were detected on the CS-(HA-AMPCol)10 surface (Fig. 3A). The S signal stands for cysteine in AMPCol.

As layers increased, N was enhanced, corresponding to an increase in HA and AMPCol (Fig. 3B). Reduced Ti signal indicated a decrease in the uncoated titanium area corresponding to greater layer number (Fig. 3C).

**Figure 4:**
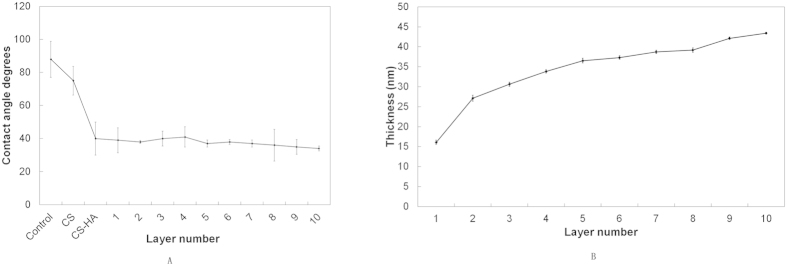
(**A**) Contact angles of the titanium surface with different deposition cycles demonstrated the wetability of the surfaces. (**B**) Thickness of CS-(HA-AMPCol)n multilayer films as a function of bilayer number were measured by QCM at 15 MHz overtones.

In order to determine the wetability of the measured substrates, contact angle measurements were taken. As shown in Fig. 4A, the contact angle of the uncoated pure titanium surface was 88°, which decreased to 75.01° on titanium assemblies with CS. Upon addition of HA, the contact angle decreased to 40.14°, and this value fluctuated between 34° and 40° as the number of coating layers increased. The wetability of the multilayered titanium films was enhanced compared to native titanium surfaces, suggesting the CS-(HA-AMPCol)n coatings were constructed successfully via the LBL technique.

We next wanted to determine the molecular thickness of the coatings on titanium surfaces. Film thickness of (HA-AMPCol)n multilayers was measured by QCM, and, as shown in Fig. 4B, the film thickness of (HA-AMPCol)n multilayers increased with deposition number. The thickness growth rate was approximately 2.2 nm per bilayer. These results indicated that the thickness of HA-AMPCol multilayer films can be precisely controlled by changing the number of deposition cycles.

**Figure 5:**
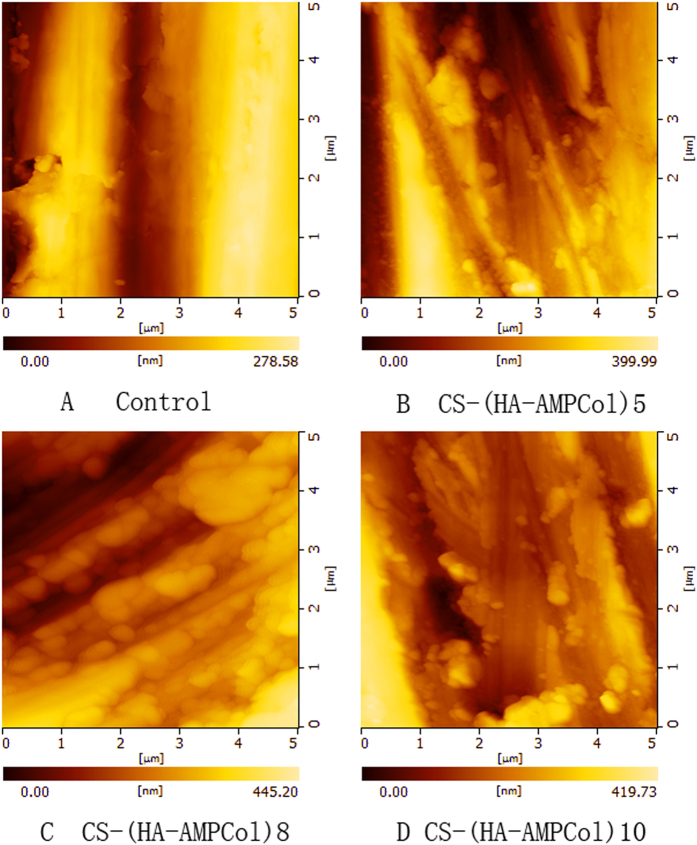
The morphologic images of uncoated titanium surface and Ti plates with CS-(HA-AMPCol)n coatings were observed by AFM: (**A**) uncoated titanium surface, (**B**) CS-(HA-AMPCol)5 coating, (**C**) CS-(HA-AMPCol)7 coating, (**D**) CS-(HA-AMPCol)10 coating.

**Figure 6:**
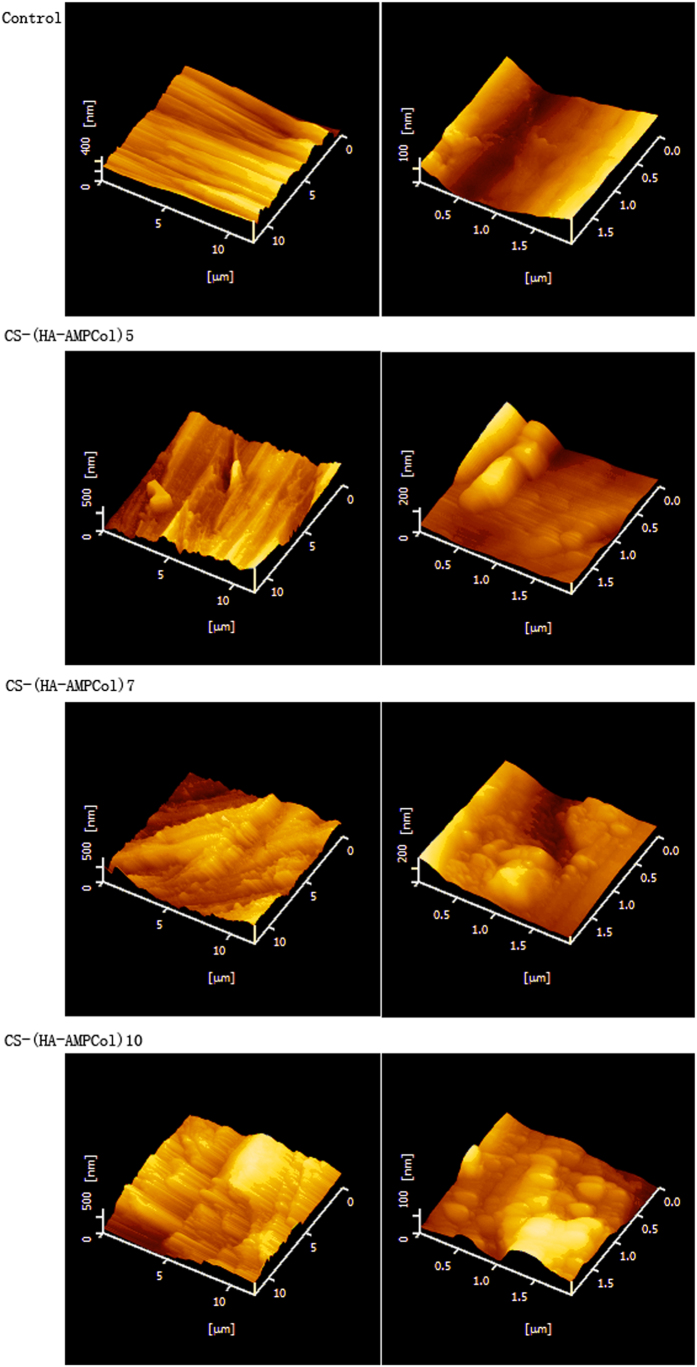
AFM 3D images of Ti plates with and without coatings: (**A**) uncoated titanium surface, (**B**) CS-(HA-AMPCol)5 coating, (**C**) CS-(HA-AMPCol)7 coating, (D) CS-(HA-AMPCol)10 coating. The right panel of each group shows the higher-resolution image.

We next wanted to characterize the surface topography of CS-(HA-AMPCol)n. Using AFM parallel grooves produced during the grinding process on the uncoated titanium surface were evident and, with increasing layers of CS-(HA-AMPCol) applied to the surface, increasing levels of surface irregularity became evident (Fig. 5). Three-dimensional images shown in Fig. 6 allowed visualization of the CS-(HA-AMPCol)n assembly on the titanium surface with granular-like structures at lower resolution (Fig. 6B–D, left) which appeared to be island-like structures at high resolution (Fig. 6B–D, right).

The titanium with CS-(HA-AMPCol)5 (14.17 ± 0.15 MPa) had the highest values of tensile bond strength when compared to the other groups. The titanium with CS-(HA-AMPCol)7 (14 ± 0.26 MPa) was higher than CS-(HA-AMPCol)10 (13.13 ± 0.15 MPa). There was no significant difference between these groups.

### AMPCol coated titanium surfaces results in little cytotoxicity, erythrocyte hemolysis and immunotoxicity

**Figure 7:**
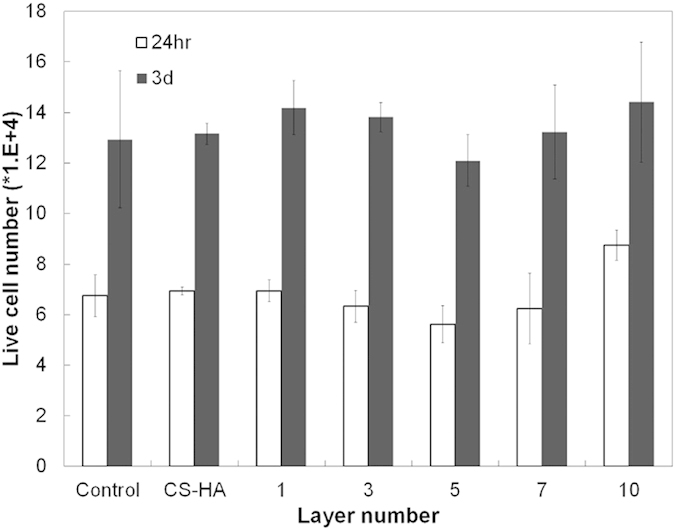
AlamarBlue assay was performed to evaluate the cytotoxicity of HaCaT cells in the solution. No statistical difference in cell viability between the CS-(HA-AMPCol)n and the two controls (Ti and CS-HA coating) after 3 days incubation was observed.

We wanted to investigate potential cytotoxic or cell damaging effects of our coated surfaces. To measure cell cytotoxicity, HaCaT cells were incubated with our coated plates and viability measured by AlamarBlue assay at 24 hours and 3 days post incubation (Fig. 7). A statistical analysis of variance showed no significant difference (p > 0.05) between samples (CS-(HA-AMPCol)n) and controls (cells cultured on pure titanium plates without coating and titanium plates coated with CS-HA). Importantly, the multilayer coating did not affect viability or cellular metabolism.

**Figure 8:**
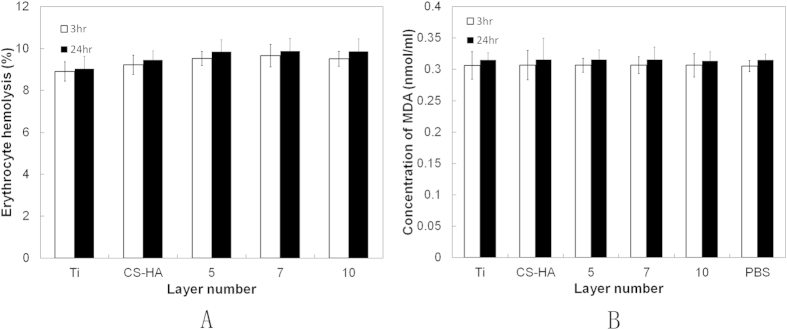
The effects of CS-(HA-AMPCol)n on erythrocyte hemolysis were tested at 3 hr and 24 hr. (**A**) Changes in MDA content in erythrocytes (**B**). The results are presented as mean ± SD, n = 3. No significant difference between the groups was observed.

Erythrocyte hemolysis was used as a valuable marker of erythrocyte membrane damage, which causes the release of hemoglobin and other cellular components into the extracellular environment. As shown in Fig. 8A, no statistical difference was observed between coated and uncoated conditions (p > 0.05) neither in 3 hr nor in 24 hr, suggesting the AMPCol coating did not cause a significant increase in erythrocyte hemolysis compared with the untreated control. The values of 24 hr were higher than 3 h, without significant difference.

In order to further determine the potential effect of the multilayer coating on erythrocytes, the accumulation of MDA was investigated. Figure 8B shows that titanium plates with or without coating had comparable MDA accumulation. Importantly, in 3 hr and 24 hr, no significant differences between our titanium surfaces and PBS control condition were observed (p > 0.05).

To determine if our coatings resulted in immunotoxicity *in vivo*, we injected AMPCol amount into BALB/c mice for 28 days and then examined spleen weight and serum immunoglobulin levels. The AMPCol amount was the average amount released from the coating per day.

**Figure 9:**
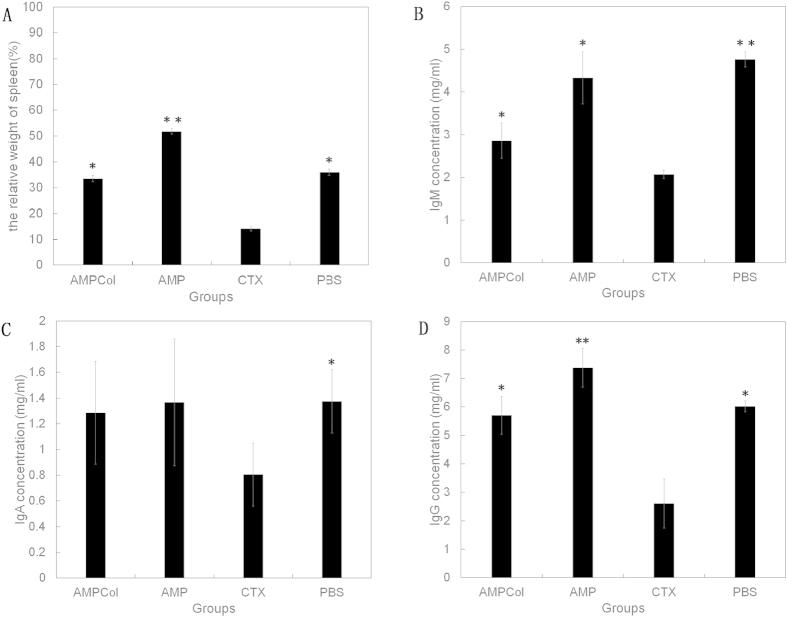
Effects of AMPCol and AMP on the levels of the relative weight of spleen (**A**), IgM (**B**), IgA (**C**) and IgG (D) from the serum were measured using ELISA. The data represent mean ± SD, *p < 0.05, **p < 0.01.

The effects of AMPCol and AMP on weight of the spleen were examined. The CTX significantly decreased the relative weight of the spleen. However, AMPCol and AMP had no effects on the weight of the spleen (Fig. 9A).

As shown in Fig. 9B–D, we compared immunoglobulin (Ig) serum levels of mice injected with AMPCol, AMP, CTX or PBS to assess the systemic immune response. There were significant decreases in levels of IgG, IgM, and IgA in CTX-treated mice compared to the control-treated mice. However, no significant difference was observed in Ig serum levels between control mice and those treated with either AMPCol or AMP.

### Sustained Antimicrobial Activity and Biofilm Inhibition

**Figure 10:**
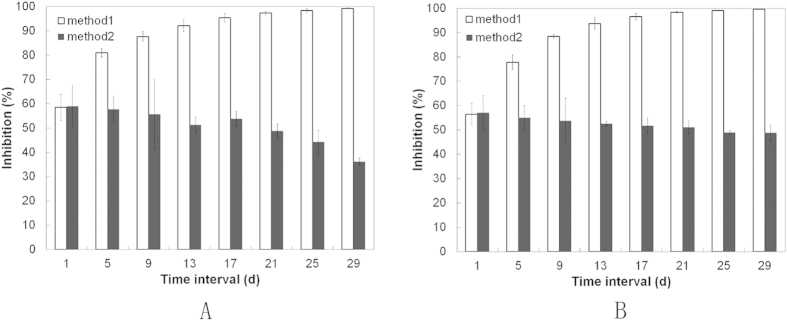
Sustained antimicrobial activity showed that *P. gingivalis* (**A**) and *S. aureus* (**B**) efficiently inhibited by CS-(HA-AMPCol)10 samples for up to one month. Samples were collected by two methods, collecting part of the broth without refilling (method 1), and gathering all the broth then adding an equal amount of broth (method 2).

Finally, we wanted to determine if sustained antimicrobial activity could be obtained with our AMPCol multilayer coating against *P. gingivalis* and *S. aureus*. We collected samples for analysis by two methods. By method 1 (collecting part of the broth without replenishing), after 24 h of culture with the CS-(HA-AMPCol)10, *P. gingivalis* and *S. aureus* were inhibited by 58.5% and 56.4% respectively, relative to controls (Fig 10). Similarly, by method 2 (collecting all broth for analysis then adding an equal amount of fresh broth), *P. gingivalis* and *S. aureus* were inhibited by 59% and 57.07%, respectively. As shown in Fig. 10, *P. gingivalis* and *S. aureus* were inhibited by 99.31% and 99.56%, respectively over 29 days by inhibition method 1, and by 36.17% and 48.8%, respectively, by inhibition method 2.

**Figure 11:**
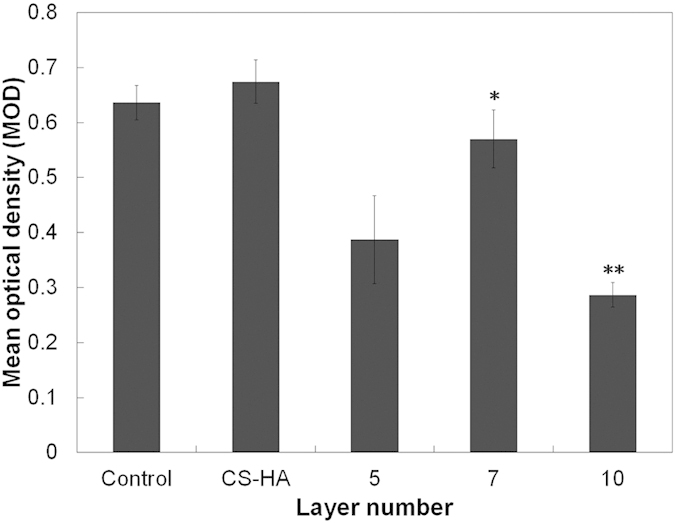
Early biofilm formation (MOD) of *S. aureus* grown on the titanium plates with and without coating was tested. CS-(HA-AMPCol)7 and CS-(HA-AMPCol)10 exerted a significant inhibitory effect on biofilm formation (p < 0.05). The data represent mean ± SD, *p < 0.05, **p < 0.01.

We next wanted to investigate the effects of our coatings on early biofilm formation. As an indicator of biofilm formation, we measured MOD, which is proportional to the area of early biofilm formation, and IOD, which is related to the total amount of fluorescence present. MOD is equal to IOD divided by the area of positive staining. As shown in Fig. 11, the MOD of the CS-HA group was not significantly different than the uncoated titanium plate (p = 0.31). The MOD of CS-(HA-AMPCol)5 was insignificantly less than the uncoated titanium (p = 0.0607), but the MOD in the other two coatings was significantly less than the control (p < 0.05). These results indicated the thicker multilayer coatings exerted a strong inhibitory effect on the early biofilm formation of *S. aureus*, specifically CS-(HA-AMPCol)7 and CS-(HA-AMPCol)10.

## Discussion

With an increasing prevalence and limited available therapies, peri-implantitis has become one of the hottest research topics. Peri-implantitis starts from the soft tissue at the implant neck, which is the boundary between hard and soft tissue. One strategy for preventing peri-implantitis is to establish an antimicrobial coating on the implant neck. The ideal coating should have three essential properties: (1) inhibit all pathogens of peri-implantitis, (2) have few cytotoxic effects, and (3) sustain drug release throughout the healing period after implant placement. In this study, we developed an antimicrobial multilayer coating on the smooth titanium surface using the LBL technique to fulfill the requirement for these properties. Implants with these coatings on their necks can limit bacterials growing after placement.

XPS wide scans of assembled surfaces showed that Ti, O, C, N and S were the most prevalent elements of the coated surface composition corresponding to the constituents of the AMPCol coating. The significantly increased signal intensity of N in the multilayer films positively correlating with additional layers may be due to HA and AMPCol amine groups, while the corresponding decrease in the Ti peak may be due to the uncoated area of the titanium surface becoming smaller as the adsorption of polyelectrolytes form a thin coating film. AFM images showed increased island-like structures on the titanium plates following the addition of the bilayer coating. Hydrophilicity of the coated plates increased, which may play an important role in cell-surface interactions^53^. These results demonstrated that the CS-(HA-AMPCol)n coatings were successfully adsorbed by the polished titanium plates. The results of QCM indicated that deposition process was cumulative through 10 cycles although the thickness growth rate before the fifth cycle was higher than the rate of accumulation after the sixth cycle (Fig. 4B). Deviations in the growth rate measurements here may have been due to a limited quartz crystallization area. Additionally, by the fifth cycle, the charges on the crystal were close to saturation resulting in subsequently weaker layer attachment and a decreased thickness growth rate. Collectively, these results suggest the thickness of the coating can be controlled using the LBL technique. The QCM results might be related to the results of mechanical property test, which showed that the CS-(HA-AMPCol)5 titanium plates had the highest tensile bond strength. Compared with normal denture relining materials^46^, all the coatings had much better tensile bond strength. With the increase of the coating thickness, it might be more easily peeled. Since this coating is expected to be located at the neck of dental implant, which will not be screwed into the bone, the tensile bond strength is more important than the shear bond strength. These results showed that this coating had an ideal tensile bond strength.

If biomaterials for potential applications are to be in contact with blood, they must not cause erythrocyte destruction. Hemolysis is used as an acute toxicity assay to evaluate the compatibility of materials with erythrocytes^54^,^55^. Therefore, the multilayer coating-erythrocyte interaction was evaluated by determining the extent of disruption of the erythrocyte membrane, allowing a direct measure of the toxicity of the coating. As erythrocyte hemolysis testing and the MDA results showed, the AMPCol coating did not produce hemolysis. The results of 3 hr and 24 hr showed that this coatings were stably in blood. The wetability of the surface is usually accepted as one of the main parameters affecting the erythrocyte compatibility of a coating and, taken together, our findings corroborated this^56^,^57^. To assess the cytotoxicity of our coating, HaCaT cells were incubated with coated plates to mimic the implant environment. Cell viability differed little between coated surfaces versus control plates, indicating the AMPCol coating is not toxic to cells. Improved cell viability in our system may also be attributed to an increase in wetability and the presence of collagen IV, which is present in basement membranes of human tissues in the internal basal lamina of the peri-implant epithelium. Collagen IV is also an important component in the interaction of cells with the underlying basement membrane, and this interaction is critical for various biological processes, such as cell adhesion, migration, proliferation and differentiation. Murray *et al.* demonstrated that collagen IV is the binding substrate for keratinocytes^58^. Collectively, we hypothesize the presence of collagen IV makes our AMPCol coating biocompatible for epithelial cells.

Immunotoxicity assays are important tests for new drugs and other biomaterials developed for application in the human body. Considering the complexity of the immune system, *in vivo* studies are more physiologically relevant. CTX is a nitrogen mustard alkylating agent from the oxazaphosphorine group and is used to treat cancers and autoimmune disorders. It interferes with DNA replication by forming intra- and interstrand DNA crosslinks. CTX, which was chosen as the positive control in this study, is a broad spectrum cell cycle inhibitor, so one of its side effects is immunosuppression^59^,^60^. The spleen has been found to be one of the most important lymphoid organs involved in the active immune response^61^,^62^ and is sensitive to damage by xenobiotics^63^,^64^. In the present study, relative spleen weight decreased in mice treated with CTX relative to the control group, but significant differences were not observed between mice treated with AMPCol or AMP and the PBS-treated mice. IgM, IgA and IgG are the dominant immunoglobulins in the serum and their levels are used as important indices of host humoral immune function^65^. IgG is the main type of antibody found in blood and extracellular fluid allowing it to control infection of body tissues^66^. IgA plays a critical role in mucosal immunity and can inhibit inflammatory effects of other immunoglobulins^67^,^68^. IgM, produced by the spleen, is the first antibody to appear in response to initial exposure to an antigen and can activate complement^69^,^70^. Our results indicated that CTX suppressed humoral immune function as serum Ig levels in CTX-treated mice were significantly lower than in untreated animals. In contrast, mice that underwent prolonged IP administration of either AMPCol or AMP had comparable immunoglobulin levels to untreated animals. According to other studies^50^,^71^, these results suggest that AMPCol and AMP effect on immune system function on the parameters determined.

The most important function of the coating used to prevent peri-implantitis is sustained antimicrobial activity. We sampled broth with AMPCol every 4 days by either (1) collecting part of the broth or (2) gathering all the broth and subsequently replenishing an equal amount of fresh broth. Method 1 represented the total cumulative release of AMPCol, while method 2 showed AMPCol release over 4 days. The healing period for soft tissue after implant placement is approximately one month, therefore the degradation test and sustained antimicrobial assay were carried out over 28 days. When considering the initial burst release, more time points were included on the first day, including 0.5 h, 1 h, 3 h and 5 h. Generally, broth obtained using method 1 contained more AMPCol than using method 2. Importantly, our approach in method 1 most accurately reflects the *in vivo* environment. A biofilm is described as a relatively undefinable microbial community associated with the tooth surface or implants. Bacterial cells colonize the tooth surface almost immediately after cleaning, within 4 hours of the pellicle formation. The early colonizers are mainly Gram-positive cocci, rods, and actinomyces^72^. Acting as the substrate, these biofilms evolve and allow other microorganisms to attach and multiply on the surfaces. Inhibiting early biofilm formation can control bacterial accumulation and infection of the peri-implant tissues. In the present study, *S. aureus*, a Gram-positive cocci, was stained using acridine orange, a nucleic acid selective fluorescent cationic dye. MOD, the mean optical density, is directly proportional to the amount of biofilm. Our results suggested that the amount of biofilm was inversely proportion to the number of coating layers and that the 10 layered AMPCol is highly effective at inhibiting *S. aureus* biofilm formation. Collectively, these data demonstrated that we had developed an antimicrobial sustained release system using the LBL technique that inhibited bacterial growth for almost a month. The next step is *in vivo* comparison studies against Peridex, tetracycline fibers, or Minocycline Hydrochloride Ointment.

## Conclusion

A multilayer coating containing an antimicrobial peptide was fabricated on smooth titanium via the LBL assembly process. The AMPCol-loaded layers formed a thin hydrophilic film. The surface encouraged cellular attachment with low levels of cytotoxicity or erythrocyte hemolysis. *In vivo* exposure to AMPCol and AMP did not cause apparent significant damage to systemic immunity, which indicated high immunocompatibility. A sustained release assay and antimicrobial tests showed that the titanium plate with AMPCol coating enabled sustained antimicrobial activity and inhibited early biofilm formation. Overall, this new multilayer coating demonstrated excellent long-term sustained antimicrobial activity.

## Additional Information

**How to cite this article**: Shi, J. *et al.* Biological and immunotoxicity evaluation of antimicrobial peptide-loaded coatings using a layer-by-layer process on titanium. *Sci. Rep.* **5**, 16336; doi: 10.1038/srep16336 (2015).
